# Patient-reported Outcomes and 3-dimensional Surface Imaging after Risk-reducing Mastectomy and Immediate Breast Reconstruction

**DOI:** 10.1097/GOX.0000000000003561

**Published:** 2021-05-21

**Authors:** Lucy Bai, Kerstin Sandelin, Marie Wickman, Brita Arver, Ola Lundström, Hemming Johansson, Yvonne Brandberg

**Affiliations:** From the * Department of Oncology-Pathology, Karolinska Institutet, Stockholm, Sweden; † Department of Molecular Medicine and Surgery, Karolinska Institutet, Stockholm, Sweden; ‡ Department of Medical Imaging, Karolinska University Hospital, Stockholm, Sweden; § Sophiahemmet University, Stockholm, Sweden.

## Abstract

The cosmetic results after risk-reducing mastectomy (RRM) and immediate breast reconstruction (IBR) are intended to be long-lasting. Long-term follow-up of the cosmetic outcome can be evaluated subjectively by the women themselves through patient-reported outcome measures such as questionnaires, or by using data from three-dimensional surface imaging (3D-SI) to calculate the volume, shape, and symmetry of the reconstructed breasts as a more objective cosmetic evaluation. The study aim was to evaluate the correspondence between patient-reported measures and 3D-SI measurements.

**Methods:**

Questionnaires (EORTC QLQ-BRECON23 and BIS) were sent to women on average 13 [7–20] years after RRM and IBR. Items were preselected for comparison with 3D measurements of women imaged using the VECTRA XT 3D-imaging system at the long-term follow-up.

**Results:**

Questionnaire responses and 3D images of 58 women, 36 without and 22 with previous breast cancer (where 15 also received radiotherapy) before RRM and IBR, were analyzed. Median age at follow-up was 57 [41–73] years. Patient-reported satisfaction with the cosmetic outcome was positive for both groups. 3D measurements indicated more symmetrical cosmetic results for women without previous breast cancer. No statistically significant associations between patient-reported satisfaction and 3D measurements were found.

**Conclusions:**

Satisfaction with the long-term cosmetic outcome after RRM and IBR was, in general, positive when evaluated by the women. 3D-SI could be used as a more objective approach to assess the cosmetic outcome in terms of volume and shape-symmetry; however, it does not directly translate to the patient-reported satisfaction.

## INTRODUCTION

Long-term follow-up studies are warranted for women with increased hereditary risk for breast cancer considering risk-reducing mastectomy (RRM) and most often immediate breast reconstruction (IBR). The majority of these women are young and otherwise healthy and should not only be informed about the possible short-term impact of the surgery on their psychosocial health, but also the long-term effects.^1,2^ Since the cosmetic results are supposed to be long-lasting, long-term follow-up of women going through risk-reducing surgery may add valuable information to women who are considering the operation.

The cosmetic outcome after breast reconstruction can be evaluated by the patients themselves through self-reported questionnaires,^3–6^ or can be assessed by a panel of external observers who analyzes 2-dimensional photographs of the patients. Although the overall aesthetic result and symmetry of the breasts after RRM and IBR have been found to be scored as good by both a panel of experts and the patients themselves,^5^ subjective evaluation of aesthetic outcome is heterogenous, thus making inter- and intra-study comparisons challenging. In addition, varying quality of intra-class correlation and agreement between expert assessments have been reported.^7–9^

With a goal to minimize heterogenicity, more objective approaches for assessing the aesthetic outcome, such as the semiautomated software Breast Cancer Conservative Treatment (BCCT.core) have been examined. Although no significant difference was observed between BCCT.core and expert assessment, both approaches seem to score poorer satisfaction with the cosmetic results than the patients scored themselves.^8,10^

Three-dimensional surface-imaging (3D-SI) methods are now being introduced in the clinic to evaluate the aesthetic outcome after breast surgery more objectively.^10,11^ However, the use and development of 3D-SI technology have mainly focused on the surgeon’s perspective.^12^ There is a lack of studies comparing the evaluation of the aesthetic outcome after RRM and IBR using 3D-SI techniques compared with the patients’ own report. The aim of this study was to use validated questionnaires and 3D-SI to evaluate the long-term cosmetic results after RRM and IBR, and to investigate associations between patient-reported outcome measures and 3D-SI measurements.

## METHODS

### Participants and Procedure

All women had a verified family history of breast cancer, and the majority carried a confirmed *BRCA1*- or *2*-gene mutation. They underwent RRM and IBR with submuscular permanent silicone implants between 1997 and 2010 at Karolinska University Hospital, Stockholm. Most women were asymptomatic and underwent bilateral RRM. Some women had been treated for breast cancer before the RRM and underwent a contralateral RRM and an ipsilateral complementary RRM if breast conserving surgery had been performed. All women underwent bilateral IBR. Surgical procedures have previously been described in detail.^5,13,14^ The inclusion criterion was participation in the short-term prospective psychosocial follow-up studies, *ie*, having responded to questionnaires at least at 1 time point (out of 4) during the 2-year follow-up period.^15–17^ The exclusion criterion was any type of cancer diagnosis post-RRM (recurrence or de novo).

During the winter of 2016, eligible women were contacted via paper letter. The letters contained an information sheet explaining the purpose and design of the long-term follow-up study, an informed consent form asking for permission for 3D-SI and to view their medical records to update the information of their clinical data, psychosocial questionnaires to be completed, an invitation for 3D-SI, and a prepaid return envelope. A reminder was sent to non-responders after 1 month. Questionnaire responses and signed informed consent forms were returned to, registered, and archived by the study coordinator (LB). Data collection of questionnaire responses proceeded until May 2017.

Subsequently, reserved dates for 3D-SI were sent to those who had accepted the invitation. Images of the women were captured using the VECTRA XT 3D-imaging system (by Canfield Sci, N.J.). This 3D-SI system uses stereophotogrammetry to estimate *x*, *y*, *z* coordinates of the imaged surface. Data collection of 3D surface images proceeded until February 2018. All images were coded to a key to preserve anonymity before the data analysis that was performed using the computer software VECTRA Analysis Module. Data analysis of all images was completed in April 2019. In a previous methodological study, 3D measurements obtained by 2 independent observers (OL and LB) were compared.^18^ It was found that the inter-observer reproducibility was moderate, the intra-observer reproducibility was good, and the inter- and intra-posture reproducibility were good. For the purpose of the current study, we included the 3D measurements analyzed by 1 observer only, obtained from one image per participant posing with their hands on their hips.

### Questionnaires

**Fig. 1.**
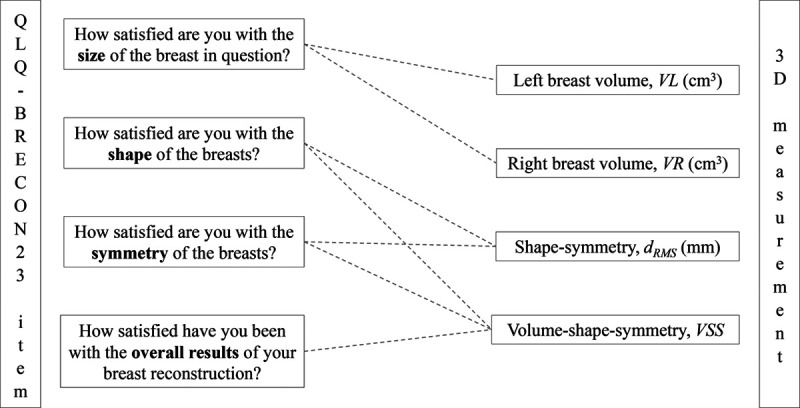
Preselected items from the EORTC breast reconstruction questionnaire module (QLQ-BRECON23) and its matching 3D measurement(s).

The European Organisation for Research and Treatment of Cancer (EORTC) Quality of Life Questionnaire Module (QLQ-BRECON23) is designed for breast cancer patients after breast reconstruction, assessing postoperative satisfaction with the results.^19^ The Swedish translation has been validated and reliability tested.^20,21^ The questionnaire consists of 23 items, with the scores 1 =“Not at all,” 2 =“A little,” 3 =“A lot,” and 4 =“Very much.” In the present study, specific items were preselected to correspond to the 3D measurements obtained from 3D surface images (Fig. 1).

The body image scale (BIS) measures the impact of surgery on self-consciousness, physical and sexual attractiveness, femininity, satisfaction with body and scars, body integrity, and avoidance behavior after surgery.^22^ It consists of 10 items, with the scores 0 =“Not at all” to 3 =“Very much” per item. The higher the total BIS score [range 0–30], the more problems.

### 3D-SI measurements

**Fig. 2.**
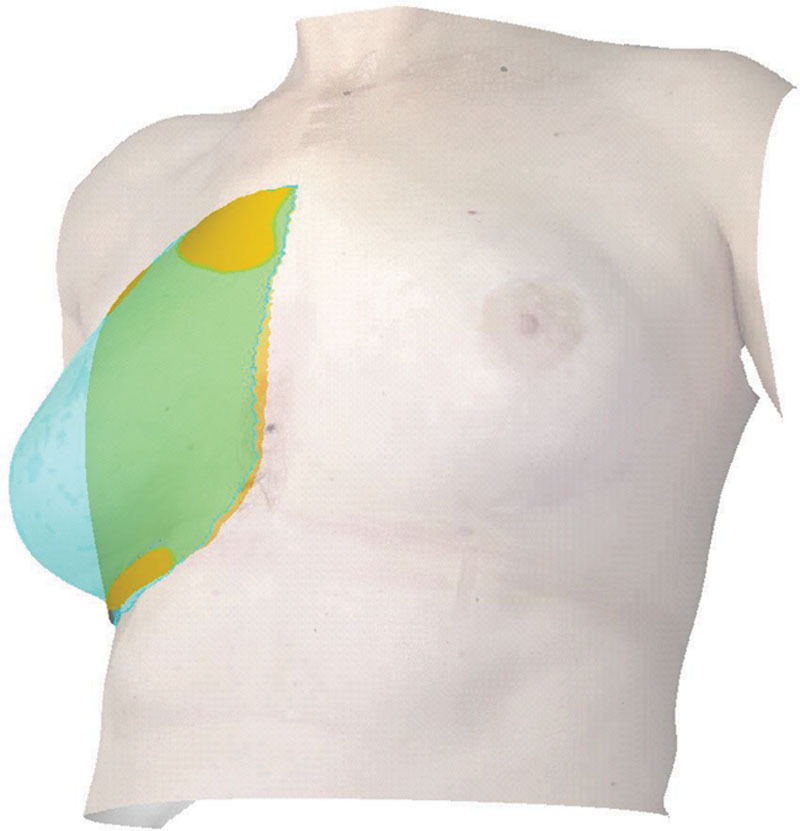
Snapshot during breast volume analysis of a 3D surface image in VECTRA Analysis Module, demonstrating an interpolated virtual chest wall (yellow) visible under the translucent surface of the reconstructed right breast (turquoise).

Breast volumes of the left *(VL*) and right breast *(VR*) were calculated in VECTRA Analysis Module through interpolation of a virtual chest wall as the back boarder of the imaged breasts’ surface (Fig. 2). The volumes were expressed in cubic centimeters (cm^3^).

**Fig. 3.**
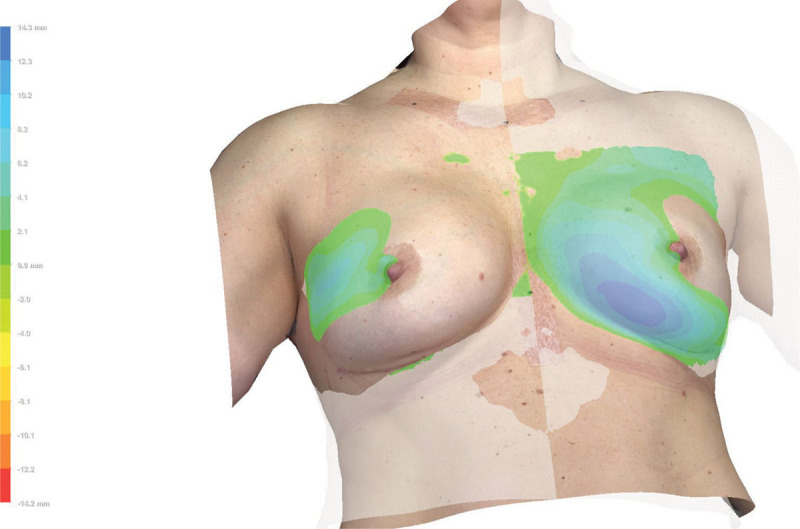
Snapshot during shape symmetry analysis of a 3D surface image in VECTRA Analysis Module, demonstrating a mirrored copy of the original breast surface area superimposed and layered above its original surface, aligned where *x* = 0. Differences in distances (mm) between the coordinates of the breast surface areas are enhanced using color gradients (to the left).

Shape symmetry *(d*_*RMS*_) was calculated from the square root of the mean distances between the coordinates of an image of the breast surface and the surface of its mirror image superimposed over the original surface squared (Fig. 3). It is a way of expressing how symmetrical the left and the right sides of the torso are, as the mean distances between the surfaces are calculated based on corresponding coordinates on the 2 surfaces. The closer the number is to 0, the more symmetrical is the torso. The magnitude of *d*_*RMS*_ (expressed in millimeters) and its clinical implications have, however, not been quantified yet.

Volume-shape-symmetry *(VSS*) is a unitless parameter that combines the volume measurements with *d*_*RMS*_. It ranges from 0 to 1, where 1 can be interpreted as having perfectly symmetrical breasts in terms of volume and shape:


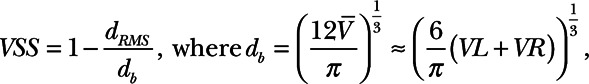


and acts as a characteristic diameter of the breasts, and 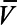 is the mean volume of the left and right breast by assuming the breast volumes as geometrical estimates.^18^

### Ethical Approval

The study was approved by the Regional Ethics Committee in Stockholm (dnr 2015/735-31/4).

### Statistical Analysis

Descriptive statistics such as counts and percentages were used for categorical data (the patient-reported outcome measures from QLQ-BRECON23 and BIS) and medians and range for continuous data (the 3D measurements *VL*, *VR*, *d*_*RMS*_, and *VSS*). Tests for associations between the continuous 3D measurements and the patient-reported outcome measures categorized into 4 different response options (1= “Not at all,” 2 =“A little,” 3 =“A lot,” and 4 =“Very much”) for the items from QLQ–BRECON23 and the total BIS score were performed using the non-parametric Kruskal-Wallis test. No power analysis was conducted specifically for this study. All statistical analyses were performed using the software STATA/IC 14.2, StataCorp, Texas. The level of statistical significance was set at 0.05.

## RESULTS

**Fig. 4.**
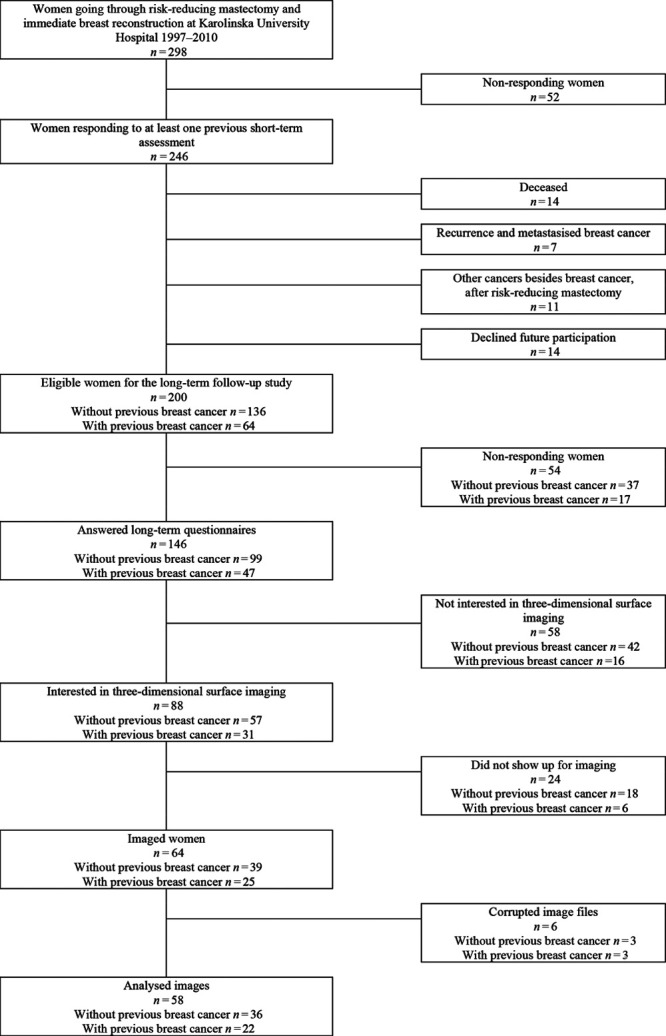
Consort diagram of eligible participants, criteria for inclusion and exclusion, and the final study participants.

In total, 88 women accepted and were invited for 3D-SI after having responded to the questionnaires (Fig. 4).^2^ Sixty-four of them (73%) participated in 3D-SI. The image files of six (9%) women were corrupted and thus excluded. For the remaining 58 women (22 with and 36 without previous breast cancer), the median age at follow-up for 3D-SI was 55 (min–max 41–73) years (Table 1). The median number of years since surgery was 12 (min–max 7–20) years. The median and mean time between responding to the questionnaires and 3D-SI was 11 (min–max 5–23) months. None of the participating women had ptotic breasts.

**Table 1.** Clinical Data of Study Participants

Variable	Cancer	No Cancer
	n (%)	n (%)
No. women	22	36
Age at 3D surface imaging (y)		
40–49	3 (14)	10 (28)
50–59	8 (36)	16 (44)
60–69	5 (23)	8 (22)
≥70	6 (27)	2 (6)
*BRCA* mutation status		
*BRCA1*	8 (36)	15 (42)
*BRCA2*	3 (14)	8 (22)
BRCAX^*^	10 (45)	9 (25)
Missing	1 (5)	4 (11)
Type of breast cancer		
In situ	4 (18)	
Invasive	17 (77)	
Missing	1 (5)	
Type of breast surgery		
Risk-reducing mastectomy	22^**†**^ (100)	36 (100)
Immediate implant-based breast reconstruction	22 (100)	33 (92)
Missing		3 (8)
Radiotherapy		
Yes	15 (68)	
No	6 (27)	
Missing	1 (5)	
Chemotherapy		
Yes	15 (68)	
No	5 (23)	
Missing	2 (9)	
Endocrine therapy		
Yes	11 (50)	
No	7 (32)	
Missing	4 (18)	
Reoperations after risk-reducing mastectomy		
Planned^‡^	8 (36)	16 (44)
Unanticipated^§^	14 (64)	17 (48)
Missing		3 (8)

^*^BRCAX = women with breast cancer and/or ovarian cancer, screened negative for *BRCA1* and *BRCA2*, but with family history of breast cancer.

^†^Number of women undergoing complementary/contralateral mastectomy after breast cancer surgery: *n*(breast conserving surgery) = 10, *n*(mastectomy) = 12.

^‡^Removal of filling port, nipple reconstruction.

^§^Unanticipated surgeries after RRM and IBR requiring general anesthesia, *eg*, implant-related issues, immediate postoperative complications, aesthetic concerns.

### Patient-reported Satisfaction

**Fig. 5.**
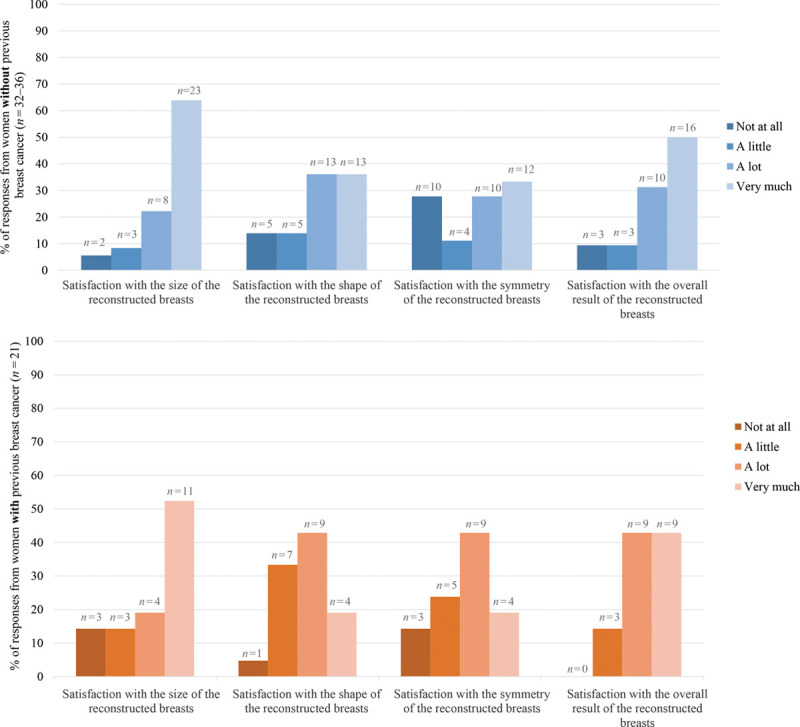
Distribution (%) of responses to items from the EORTC breast reconstruction questionnaire module (QLQ-BRECON23) regarding satisfaction with the reconstructed breasts in terms of size, shape, symmetry, and overall result, for women without (top) and with (bottom) previous breast cancer prior RRM and IBR. The non-responders were excluded: women without previous breast cancer not responding to the item about satisfaction with the overall result *n* = 4; women with previous breast cancer not responding to either of the four items *n* = 1.

Figure 5 shows that the majority of women without previous breast cancer responded that they were “A lot” or “Very much” satisfied with the size, shape, symmetry, and overall result of their breast reconstruction. Similar distributions were seen for women with previous breast cancer. The lowest level of satisfaction for women without previous breast cancer was for breast symmetry. In general, women with previous breast cancer reported lower satisfaction regarding shape and symmetry compared with the satisfaction with breast size and overall result.

**Fig. 6.**
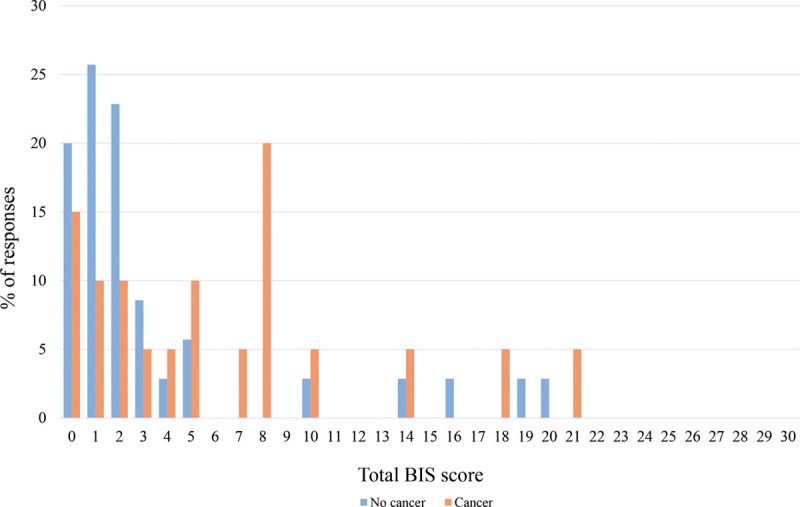
Total body image scale (BIS) scores and the distribution (%) of responding women without (*n* = 35) and with (*n* = 20) previous breast cancer. The non-responders were excluded: women without previous breast cancer *n* = 1; women with previous breast cancer *n* = 2.

The total BIS scores are shown in Figure 6. The median total BIS scores for women without and with previous breast cancer were 2.0 (min–max 0.0–20.0) and 5.0 (min–max 0.0–21.0), respectively.

### 3D Measurements

**Fig. 7.**
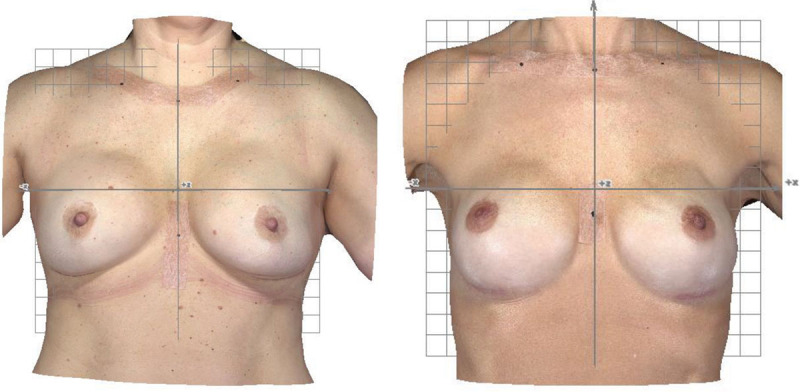
Frontal view of 3D surface images of 2 women with similar cosmetic outcome of the left and the right breast. Visible grids aligned with the jugulum–xiphoid process where *x* = 0, and jugulum –7 cm to the left and right on the clavicles aligned where *y* = 0. 3D measurements for the women to the *left*: volume of the left breast (*VL*) 455 cm^3^, volume of the right breast (*VR*) 468 cm^3^, shape symmetry (*d*_*RMS*_) 6.35 mm, and *VSS* 0.947; 3D measurements for the women to the *right*: *VL* = 303 cm^3^, *VR* = 310 cm^3^, *d*_*RMS*_ = 6.26 mm, and *VSS* = 0.941.

**Fig. 8.**
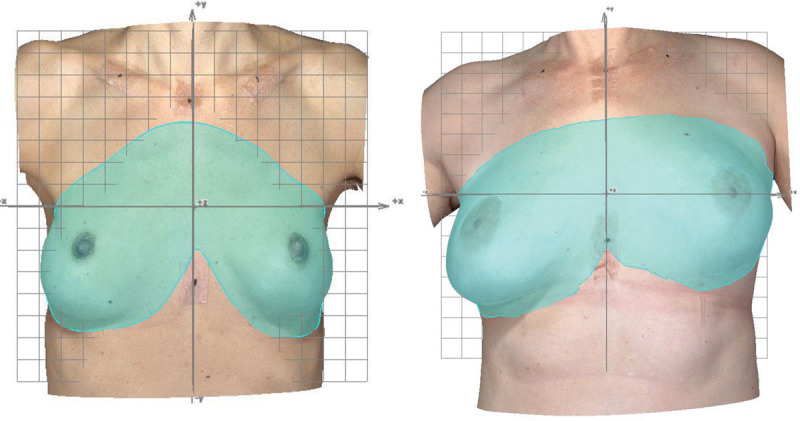
3D surface images of 2 women with varying cosmetic outcomes. The snapshots are captured during image analysis in VECTRA Analysis Module, illustrating marked out breast surface areas (turquoise) that are going to be used for shape symmetry calculations. 3D measurements for the women to the left: volume of the left breast (*VL*) 192 cm^3^, volume of the right breast (*VR*) 190 cm^3^, shape symmetry (*d*_*RMS*_) 9.24 mm, and volume-shape-symmetry (*VSS*) 0.897. 3D measurements for the women to the right: *VL* = 512 cm^3^, *VR* = 380 cm^3^, *d*_*RMS*_ = 15.7 mm, and *VSS* = 0.869.

In Figures 7 and 8, 3D surface images of women with varying cosmetic results are presented to exemplify the magnitude of 3D measurements in relation to the image viewed by the observer. The most symmetrical cosmetic results in terms of 3D measurements obtained in the cohort were *d*_*RMS*_(*lowest*) = 2.98 mm [range: 0–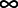] and *VSS*(*highest*) = 0.976 [range 0–1]. The median volumes of the left and right breasts of women with and without previous breast cancer were *VL*+*VR*(*cancer*) = 316 + 302 cm^3^ and *VL*+*VR*(*no cancer*) = 338 + 337 cm^3^ (Table 2). The median shape symmetry results were *d*_*RMS*_(*cancer*) = 8.49 mm and *d*_*RMS*_(*no cancer*) = 6.98 mm, and the corresponding median volume-shape-symmetry results were *VSS*(*cancer*) = 0.919 and *VSS*(*no cancer*) = 0.932.

**Table 2.** 3D Measurements of Women with and without Previous Breast Cancer, with Median (x̃), Minimum, and Maximum Measurements Presented

3D measurements	Cancern = 22	No Cancern = 36
	*x̃*(min–max)	*x̃*(min–max)
Left breast volume (*VL*, cm^3^)	316	338
	(164–614)	(70.4–830)
Right breast volume (*VR*, cm^3^)	302	337
	(169–648)	(78–849)
Shape-symmetry (*d*_*RMS*_, mm)	8.49	6.98
	(3.27–19.2)	(2.98–15.7)
Volume-shape-symmetry (*VSS*)	0.919	0.932
	(0.788–0.967)	(0.869–0.976)

### Comparison of Patient-reported Outcomes and 3D Measurements

No statistically significant associations were observed between the patient-reported outcomes and their corresponding 3D measurements (number of responses to individual items and obtained *P*-values for each preselected item and its corresponding 3D measurement are presented in Table 3).

**Table 3.** Responses to Preselected Items Regarding Breast Size, Shape, Symmetry, and Overall Result from the EORTC Quality of Life Breast Reconstruction Questionnaire Module (QLQ-BRECON23) Assessed by Women Who Have Undergone Bilateral RRM and IBR, Compared with Objective Measurements of Left Breast Volume (***VL***), Right Breast Volume (***VR***), Breast Symmetry (***d*_*RMS*_**), and ***VSS*** Obtained from 3D Surface Images of the Women 6–20 Years after the Surgery, with Median (x̃), Minimum, and Maximum Measurements Presented

QLQ-BRECON23	3D Measurement	Not at All	A Little	A Lot	Very Much	*P*
Size of the left breast (*n* = 57)	*VL x̃* (min–max) cm^3^	256 (192–491)	276 (70–449)	458 (204–645)	326 (145–830)	0.138
		(*n* = 5)	(*n* = 6)	(*n* = 12)	(*n* = 34)	
Size of the right breast (*n* = 57)	*VR x̃* (min–max) cm^3^	249 (183–499)	274 (78–334)	407 (179–648)	327 (96–849)	0.017
		(*n* = 5)	(*n* = 6)	(*n* = 12)	(*n* = 34)	
Shape of breasts (*n* = 57)	*d*_*RMS*_ *x̃* (min–max) mm	8.16 (4.33–19.2)	7.44 (2.98–17.2)	7.27 (3.92–13)	7.84 (3.44–12.9)	0.975
		(*n* = 6)	(*n* = 12)	(*n* = 22)	(*n* = 17)	
	*VSS x̃* (min–max)	0.921 (0.788–0.958)	0.934 (0.841–0.976)	0.932 (0.871–0.958)	0.919 (0.876–0.972)	0.923
		(*n* = 6)	(*n* = 12)	(*n* = 22)	(*n* = 17)	
Symmetry of breasts (*n* = 57)	*d*_*RMS*_ *x̃* (min–max) mm	7.57 (3.27–19.2)	7.97 (2.98–17.2)	7.44 (3.92–13)	7.14 (3.44–12.9)	0.885
		(*n* = 13)	(*n* = 9)	(*n* = 19)	(*n* = 16)	
	*VSS x̃* (min–max)	0.924 (0.788–0.967)	0.929 (0.841–0.976)	0.925 (0.871–0.958)	0.934 (0.876–0.972)	0.808
		(*n* = 13)	(*n* = 9)	(*n* = 19)	(*n* = 16)	
Overall result (*n* = 53)	*VSS x̃* (min–max)	0.930 (0.913–0.938)	0.939 (0.881–0.967)	0.930 (0.788–0.976)	0.924 (0.876–0.958)	0.915
		(*n*=3)	(*n*=6)	(*n*=19)	(*n*=25)	

## DISCUSSION

The patient-reported satisfaction with cosmetic outcome long-term after RRM and IBR was, in general, positive, with less body image problems among women without previous breast cancer compared with women with previous breast cancer. Sixty-eight percent of the women in the latter group had, besides having undergone a breast cancer surgery before the RRM and IBR, also received radiotherapy. The 3D measurements *d*_*RMS*_ and *VSS* indicated mathematically more symmetrical cosmetic results in terms of volume, shape, and symmetry of the reconstructed breasts for women without previous breast cancer than women with previous breast cancer. No statistically significant associations between patient-reported satisfaction and its corresponding 3D measurements were found, which can be explained by the fact that the satisfaction with the cosmetic outcome evaluated by the patient herself is a complex matter and not based only on objective measures. Consequently, although 3D measurements could potentially be used to assess the cosmetic outcome in a more objective way, they do not directly translate to the patient-reported satisfaction.

The individual backgrounds of the patients and their different experiences with losing their breasts may influence their vulnerability of, and expectations with, RRM and IBR. For approximately 60% of the women in this study, RRM and IBR was a choice made when they were healthy and asymptomatic, but conscious about their increased risk of getting breast cancer. Within this group, there might be a disparity of the dominating factors influencing their urgency to undergo RRM and IBR. For example, they might have different levels of cancer worry, anxiety and depression, or different experiences of being a close relative to a breast cancer patient with increased hereditary cancer risk. For women who had suffered from breast cancer before the RRM and IBR, the starting point is somewhat different. For instance, satisfaction with the cosmetic outcome might have been influenced by the individual cancer trajectory experiences. Similarly, scars from previous surgery and/or fibrosis and capsular contracture after radiotherapy might also have influenced the cosmetic outcome. In addition, the elapsed time since surgery and the choice of reconstructive approach have possibly affected the cosmetic outcome on its own.^23^

Satisfaction with the cosmetic outcome is often scored higher by the patients themselves compared with when it is scored by the medical staff or evaluated by using BCCT.core.^8,10,24–26^ Experts might be more trained to detect surgical and technical imperfections, while patients compare and evaluate their overall result with their preoperative condition and with their expectations.^7^ In a study investigating how well patients could predict their future satisfaction with their breasts post-mastectomy, women without breast reconstruction seemed to underestimate their future satisfaction while women with reconstructed breasts seemed to overestimate it, and misprediction was associated with regret for both groups.^27^ Therefore, the patient and the surgeon should preoperatively address the expected and realistic results of RRM and IBR. By reaching a mutual understanding, the level of satisfaction with the cosmetic results might be increased.

The patient’s own evaluation is of utmost importance when assessing the satisfaction with the cosmetic outcome. Although 3D measurements might express “good” volume, shape, and symmetry of the breasts, the woman herself might not be satisfied if these “mathematically perfect” breasts look unnatural. Nevertheless, during the data analysis process, it was clear that small differences not always detectable by the human eye could be enhanced using 3D-SI (Fig. 3), or quantified and described using the suggested 3D measurements. Therefore, the additional information provided by 3D-SI might be an aid and act as a communicational tool among surgeons or between surgeons and patients to facilitate the understanding of the implications of the surgery.

Some methodological limitations and strengths need highlighting. An inevitable part with questionnaire and invitation studies is the problem of non-responders. It is possible that a selection bias of participants interested in 3D-SI was yielded. Generalizability should be made with caution because the sample was acquired from 1 academic institution. The discrepancy in time between responding to the questionnaires and the time of 3D-SI occurred due to technical issues with the 3D-imaging system, which resulted in a delayed start of data collection of 3D surface images. However, the impact this discrepancy has on the results could be considered minimal when put into context with the long-term follow-up since the time of RRM and IBR (up to 20 years ago). Although 3D-SI is an objective instrument, the images were analyzed by one individual, which itself could implement subjective results depending on the definition of the breast area.^18^ A weakness of the software is its limited capacity to measure breast volumes of ptotic breasts as it is difficult for the program to interpolate a virtual chest wall of surfaces that are obscured, but, as none of the women in our sample had this breast shape, it was not regarded as a problem in the current study. Descriptive information was presented for both women with and without previous breast cancer before RRM and IBR to indicate differences in directions and proportions of their responses. However, no statistical analysis could be made for differences between the groups due to the small sample size.

This is, to our knowledge, the first study comparing 3D measurements with patient-reported outcomes regarding cosmetic outcome long-term after RRM and IBR. The strengths of this study were the relatively high participation rate, especially because these women were operated on average 13 years ago, which also is a strength on its own, as this longitudinal follow-up period reflects true long-term and, hence, stable cosmetic results of the surgery. Furthermore, validated questionnaires specifically designed to evaluate the satisfaction with the breast reconstruction and body image were used. To minimize the impact of systematic errors and to resemble realistic clinical conditions, 3D measurements obtained by only 1 of the 2 observers were included. In addition, this observer had the highest reproducibility scores of the 2 observers during data analysis.^18^

## CONCLUSIONS

Patient-reported satisfaction with the cosmetic outcome up to 20 years after RRM and IBR was, in general, positive. 3D-SI could be used as a more objective approach to assess the cosmetic outcome; however, it lacks a precise translatable relation with the patients’ own evaluation of the cosmetic outcome and body image as of today.

## ACKNOWLEDGMENTS

The authors acknowledge Vivianne Lindbergh, Maria Norberg Barkman, and Ingela Wåhlstrand at the Department of Medical Imaging at Karolinska University Hospital, and Farid Meybodi and Elisabeth Elder at Westmead Breast Cancer Institute, University of Sydney.
